# Effectiveness of interleukin-17A inhibitors in patients with ankylosing spondylitis: A protocol for systematic review and meta-analysis

**DOI:** 10.1097/MD.0000000000032224

**Published:** 2022-12-09

**Authors:** Mingguang Yan, Xiao Fang, Jianjun Guo, Weibing Yin

**Affiliations:** a Department of Clinical Laboratory, The First People’s Hospital of Shangqiu, Shangqiu, Henan, China.

**Keywords:** ankylosing spondylitis, interleukin-17A inhibitors, meta-analysis, systematic review

## Abstract

Ankylosing spondylitis is a chronic immune-mediated inflammatory arthritis. Interleukin-17A (IL-17A) inhibitors is recognized as a novel therapeutic target for ankylosing spondylitis. However, there is still a lack of high-quality research evidence regarding the issues. Therefore, we performed a protocol for systematic review and meta-analysis to evaluate the efficacy and safety of IL-17A inhibitors in patients with ankylosing spondylitis.

**Methods:**

This protocol will be conducted under the preferred reporting items for systematic reviews and meta-analyses protocols (PRISMA-P) guidelines. Furthermore, the study has been registered on PROSPERO (CRD42022375885). The following electronic databases will be searched regardless of language and publication status: Pubmed, MEDLINE, EMBASE, China Biomedical Database, China National Knowledge Infrastructure, VIP Database, and Wanfang Database. Cochrane “bias risk” tool is used to assess the bias risk of the quality of the included literature. Data synthesis and statistical analysis will be performed using the RevMan 5.3 (The Nordic Cochrane Centre, The Cochrane Collaboration, Copenhagen, Denmark) software.

**Results:**

A synthesis of current evidence of IL-17A inhibitors for ankylosing spondylitis will be shown in this protocol.

**Conclusion:**

This review can provide convincing evidence to help clinicians make decisions when dealing with ankylosing spondylitis.

## 1. Introduction

Ankylosing spondylitis is a chronic inflammatory disease primarily affecting the spine and sacroiliac joints.^[1–3]^ It causes pain and stiffness in the back, eventually resulting in joint damage and fusion predominantly of the sacroiliac joints, and ankylosing of the vertebrae leading to a classic bamboo spine, although this does not always occur.^[4,5]^ Besides, it can affect the rest of the axial skeleton (shoulders and hips), leading to reduced range of motion and pain. Some individuals develop joint destruction and may require total joint replacement.^[6]^ This can occur at a much earlier age than usual in individuals with ankylosing spondylitis. The disease can also cause peripheral symptoms affecting the knees, hands and feet, which can appear similar to rheumatoid arthritis, with patients experiencing pain and swelling. This can lead to misdiagnoses, thereby delaying appropriate treatment.

The disease is estimated to affect up to 1% of the general population.^[7]^ However, prevalence varies depending on race, with 0.04% to 0.06% of non-Caucasians affected by the disease compared with 0.1% to 1.4% of Caucasians.^[8]^ Ankylosing spondylitis is more common in men than in women, although the reason for this is unknown.

Although there is no cure for ankylosing spondylitis, many treatments have emerged in the management of ankylosing spondylitis in controlling inflammatory symptoms. Several lines of evidence have identified the interleukin-17 (IL-17) pathway as a promising therapeutic target in spondyloarthritis.^[9,10]^ Indeed, numbers of interleukin-17 producing cells are elevated in the circulation and target tissues in patients with ankylosing spondylitis.^[11]^ IL-17 family includes 6 members from interleukin-17A (IL-17A) to IL-17F, of which IL-17A is crucial proinflammatory cytokines. Based on a growing body of researches, IL-17A was recognized as a novel therapeutic target for ankylosing spondylitis. However, there is still a lack of high-quality research evidence regarding the issues. Therefore, we performed a protocol for systematic review and meta-analysis to evaluate the efficacy and safety of IL-17A inhibitors in patients with ankylosing spondylitis.

## 2. Methods

### 2.1. Protocol and registration

This protocol will be conducted under the preferred reporting items for systematic reviews and meta-analyses protocols (PRISMA-P) guidelines.^[12]^ Furthermore, the study has been registered on PROSPERO (https://www.crd.york.ac.uk/prospero/), registration number: CRD42022375885. The approval of the ethics committee is not necessary, because this article is a systematic review without involving the individual data of patients.

### 2.2. Inclusion criteria for study selection

#### 2.2.1. Types of studies

The study will consist of a prospective randomized controlled trials (RCTs) of IL-17A inhibitors in the treatment of ankylosing spondylitis, language of publication does not have barrier of blinding or restrictions.

#### 2.2.2. Type of participants

The patients who meet the diagnostic criteria of ankylosing spondylitis will be included, with no restrictions on age, race, disease duration, or disease severity.

#### 2.2.3. Types of interventions

The treatment group will include any type of IL-17A inhibitors such as ixekizumab, bimekizumab, netakimab, etc. Meanwhile the control group will received placebo.

#### 2.2.4. Types of outcomes

Main outcomes are 20% improvement according to the Assessment of SpondyloArthritis international Society (ASAS) criteria (ASAS20 response), and 40% improvement according to ASAS criteria (ASAS40 response).^[13]^ Additional outcomes are adverse events and quality of life.

### 2.3. Exclusion criteria

Researches with the following characteristics will be excluded: non-RCTs; studies with interventions that include other drugs besides IL-17A inhibitors; case reports, animal studies, reviews, conference papers, or RCTs with incomplete data.

### 2.4. Search strategy

The following electronic databases will be searched regardless of language and publication status: Pubmed, MEDLINE, EMBASE, China Biomedical Database, China National Knowledge Infrastructure, VIP Database, and Wanfang Database. Prospero, Clinic Trials.gov, and Google Scholar will be used to select systematic reviews or ongoing or completed clinical trials. Meanwhile, papers and bibliographies for inclusion in the trials will also be reviewed. Illustrated by the case of PubMed, the detailed search strategy is shown in Table 1, which will make appropriate adjustments according to the specific database.

**Table 1:** Search strategy in PubMed.

Number Search terms
#1 ankylosing spondylitis [Ti/Ab]
#2 spondyloarthritis [Ti/Ab]
#3 rheumatoid spondylitis [Ti/Ab]
#4 seronegative spondyloarthropathy [Ti/Ab]
#5 #1 OR #2 OR #3 OR #4
#6 anti IL-17 [Ti/Ab]
#7 interleukin 17 [Ti/Ab]
#8 IL-17 inhibitor [Ti/Ab]
#9 netakimab [Ti/Ab]
#10 bimekizumab [Ti/Ab]
#11 secukinumab [Ti/Ab]
#12 cosentyx [Ti/Ab]
#13 ixekizumab [Ti/Ab]
#14 taltz [Ti/Ab]
#15 #6 OR #7 OR #8 OR #9 OR #10 OR #11 OR #12 OR #13 OR #14
#16 #5 AND #15

### 2.5. Data collection and analysis

#### 2.5.1. Selection of studies

**Figure 1.**
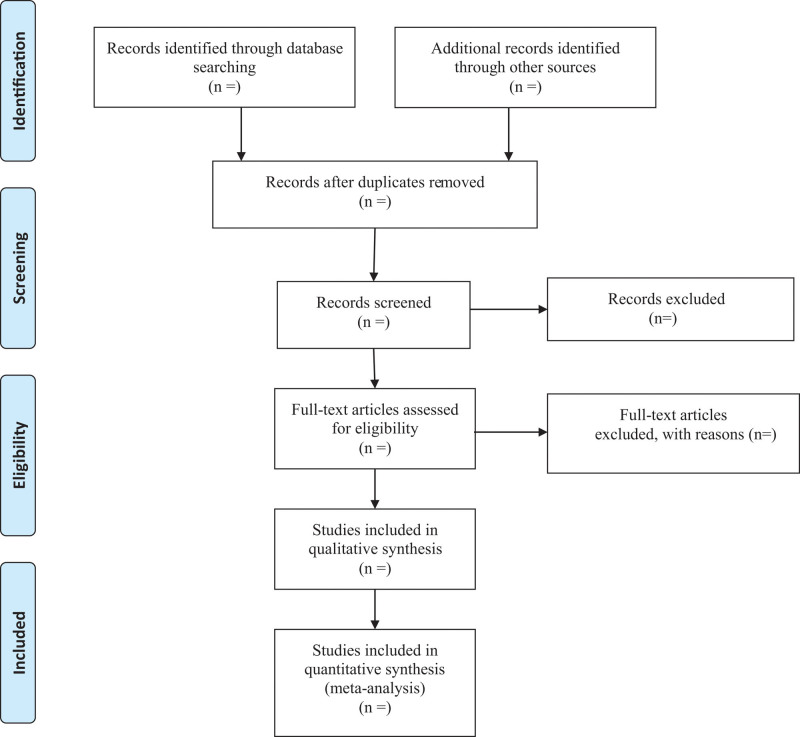
Flowchart of study selection process.

All retrieved literature that meets the requirements will be imported into Endnote X9 software (Camelot UK Bidco Limited, London, United Kingdom). Firstly, 2 researchers will independently screen the titles and abstracts to identify the potentially eligible articles. Secondly, disqualified literature will be removed based on the inclusion criteria through downloading and reviewing the full text. The reasons for exclusion should be recorded at the same time. Finally, the final eligible articles selected will be carefully cross-checked by 2 researchers. Any disagreements will be mediated via a third investigator to reach a consensus. The process of studies selection according to the PRISMA flow chart is illustrated in Figure 1.

#### 2.5.2. Data extraction

Two researchers will independently extract data according to a standard data extraction form by using Excel2013 software. The details of the form are as follows: basic information of studies (authors, title, and publication year), participants characteristics (age, gender, numbers, and course of disease), interventions, outcome indicators, adverse events, etc. If there is any incomplete information in the study, we will contact the corresponding author. All disagreements will be resolved by consulting a third researcher.

#### 2.5.3. Risk of bias assessment

Two authors will independently assess the risk of bias of the included studies based on the bias risk assessment tool recommended in the Cochrane “Risk of bias” assessment tool.^[14]^ Including 7 items: random sequence generation, allocation concealment, blind participants and personnel, blind assessment of results, incomplete result data, selective reports, and other biases. The results in each field will be divided into 3 levels: low bias risk, high bias risk, and unclear bias risk.

#### 2.5.4. Statistical analysis

Data synthesis and statistical analysis will be performed using the RevMan 5.3 (The Nordic Cochrane Centre, The Cochrane Collaboration, Copenhagen, Denmark) software. The standard mean difference with 95% confidence interval will be used to calculate the continuous data, while the dichotomous data will be measured by the rate ratio or odds ratio with 95% confidence interval. For the assessment of heterogeneity, the Chi-squared and *I*^2^ test will be carried out. If there is no significant heterogeneity among studies (*I*^2^ < 50%, *P* > .1), we will use a fixed-effect model, but a random-effects model will be employed if there exists heterogeneity (*I*^2^ ≥ 50%, *P* < .1).

#### 2.5.5. Sensitivity analysis

Sensitivity analysis will be conducted by eliminating included studies one by one and changing the statistical methods to assess the stability and reliability of analytical results.

#### 2.5.6. Grading the quality of evidence

The quality of evidence for the whole research will be assessed by the Grades of Recommendations Assessment, Development and Evaluation system,^[15]^ which was divided into 4 grades: “very low quality,” “low quality,” “medium quality,” and “high quality.”

## 3. Discussion

Ankylosing spondylitis is a chronic immune-mediated inflammatory arthritis included in the so-called group of spondyloarthritis.^[16,17]^ It typically develops in males in their third decade of life and affects mainly the axial skeleton and the sacroiliac joints. The approval of secukinumab, a fully human monoclonal antibody able to neutralize IL-17A, has been a major advance in the treatment of ankylosing spondylitis.^[18]^ Patients with ankylosing spondylitis are known to have high levels of this interleukin, which is critically involved in the pathogenesis of the disease.^[19]^

Currently, IL-17A inhibitors have not been widely used in ankylosing spondylitis due to the lack of large sample of clinical evidence. To our knowledge, this is the first meta-analysis to evaluate the efficacy and safety of IL-17A inhibitors in patients with ankylosing spondylitis. Future trials of IL-17A inhibitors should focus on populations with early disease to determine if there is indeed a better response, the effect on the progression of ankylosis in this population, and to determine when it is appropriate to start IL-17A inhibitors therapy.

## Author contributions

Mingguang Yan: finish the article; Xiao Fang: data analysis; Jianjun Guo: data collection; Weibing Yin: study design.

**Data curation:** Xiao Fang.

**Investigation:** Jianjun Guo.

**Writing – original draft:** Mingguang Yan.

**Writing – review & editing:** Weibing Yin.
